# Renal Outcomes in Children with Operated Spina Bifida in Uganda

**DOI:** 10.1155/2018/6278616

**Published:** 2018-08-07

**Authors:** Helen J. Sims-Williams, Hugh P. Sims-Williams, Edith Mbabazi Kabachelor, Benjamin C. Warf

**Affiliations:** ^1^Sheffield Kidney Institute, Sheffield Teaching Hospitals NHS Foundation Trust, Sheffield, UK; ^2^Department of Neurosurgery, Sheffield Teaching Hospitals NHS Foundation Trust, Sheffield, UK; ^3^CURE Children's Hospital of Uganda, Mbale, Uganda; ^4^Department of Paediatric Neurosurgery, Boston Children's Hospital and Harvard Medical School, Boston, MA, USA

## Abstract

**Background:**

To describe the extent of renal disease in Ugandan children surviving at least ten years after spina bifida repair and to investigate risk factors for renal deterioration in this cohort.

**Patients and Methods:**

Children who had undergone spina bifida repair at CURE Children's Hospital of Uganda between 2000 and 2004 were invited to attend interview, physical examination, renal tract ultrasound, and a blood test (creatinine). Medical records were retrospectively reviewed. The following were considered evidence of renal damage: elevated creatinine, hypertension, and ultrasound findings of hydronephrosis, scarring, and discrepancy in renal size >1cm. Female sex, previous UTI, neurological level, mobility, detrusor leak point pressure, and adherence with clean intermittent catheterisation (CIC) were investigated for association with evidence of renal damage.

**Results:**

65 of 68 children aged 10–14 completed the assessment. The majority (83%) reported incontinence. 17 children (26%) were performing CIC. One child had elevated creatinine. 25 children (38%) were hypertensive. There was a high prevalence of ultrasound abnormalities: hydronephrosis in 10 children (15%), scarring in 42 (64%), and >1cm size discrepancy in 28 (43%). No children with lesions at S1 or below had hydronephrosis (p = 0.025), but this group had comparable prevalence of renal size discrepancy, scarring, and hypertension to those children with higher lesions.

**Conclusions:**

Incontinence, ultrasound abnormalities, and hypertension are highly prevalent in a cohort of Ugandan children with spina bifida, including those with low neurological lesions. These findings support the early and universal initiation of CIC with anticholinergic therapy in a low-income setting.

## 1. Introduction

There are an estimated 37,000 children born with spina bifida every year in sub-Saharan Africa [1]. Although initial surgery can be life-saving, there is significant long-term morbidity and mortality associated with the condition, including renal complications. Chronic kidney disease (CKD) develops as a consequence of elevated detrusor pressures, vesicoureteric reflux, and recurrent urinary tract infections, leading to renal scarring [2, 3]. Additionally, urinary incontinence makes independence and socialisation more challenging and has been shown in some studies to negatively affect quality of life and self-esteem [4, 5]. In spite of the scale of the problem, there have been no publications reporting long-term renal outcomes from a low-income setting.

Modern management of the neurogenic bladder has improved outcomes, most notably with the introduction of clean intermittent catheterisation (CIC) [6]. Urological surgery, dialysis, and transplantation are not available for the vast majority of children in sub-Saharan Africa. Prevention of CKD is therefore the only option, and, to this end, CIC with anticholinergics represents the mainstay of management.

Spina bifida repair has been performed at CURE Children's Hospital of Uganda (CCHU) since 2000. Ten-year survival of Ugandan children following repair of spina bifida has been reported elsewhere [7]. The aim of this cross-sectional study was to determine the extent of renal complications in surviving children. We also investigated predictors of poor renal outcomes to determine whether children at higher risk might be identified.

## 2. Patients and Methods

### 2.1. Study Design and Participants

Eligible patients were identified from the CCHU electronic database according to the following criteria: diagnosis of open spina bifida, presentation between December 2000 and December 2004, and age younger than six months at the time of primary operative closure. To facilitate home visits, the study area was restricted to 16 local districts [7].

Families of all patients recorded as alive were telephoned or visited at home by a research assistant. Surviving children were invited to attend CCHU on a specified date accompanied by a caregiver. They were offered reimbursement of travel costs, provision of meals, and overnight accommodation if required.

Ethical approval was granted by the Institutional Review Board of CCHU.

### 2.2. Management of Neurogenic Bladder

A basic cystometric test assessing detrusor leak point pressure (DLPP) was conducted shortly after spina bifida repair, or at the first follow-up visit. A DLPP greater than 40cm H_2_O was considered to reflect a high risk bladder [8, 9]. Renal tract ultrasound scan was performed at the first follow-up appointment and on all subsequent visits.

The decision to commence CIC was based on the DLPP at initial cystometric evaluation, evidence of renal tract ultrasound abnormalities (predominantly hydronephrosis), and incontinence and its complications, such as pressure sores. Caregivers were trained in the technique of CIC, and a free supply of catheters and the anticholinergic oxybutynin (usually administered intravesically [10]) were provided by the International Federation for Spina Bifida and Hydrocephalus (IFSBH) to families at every visit.

### 2.3. Data Collection

The following information was obtained from the electronic database and clinical notes: results of cystometric testing; results of ultrasound scans; date of CIC initiation (and reason if given); documentation of urinary tract infections (UTI) with corresponding urine culture results; and comments relating to adherence with CIC. UTI was defined as “febrile or otherwise symptomatic with a positive urine culture” [11].

Informed consent was obtained from all caregivers, and children also completed an assent form. A translator was used in the majority of cases. Separate interviews were conducted with parent and child, covering a range of topics, including continence and CIC. Where there was disagreement between child and caregiver responses; for example, relating to continence, the “worst” response was selected.

Children underwent a physical assessment, including weight, height (where possible), and neurological examination to determine best motor level. Arm span was measured in all children using a tape measure, with the child's back against the wall and arms fully extended at right angles to the trunk. Values for height and arm span were recorded to the nearest millimetre. Height-for-age and BMI-for-age percentile for each child were calculated using* WHO AnthroPlus v1.0.4*.

Blood pressure was measured on the right arm with appropriate cuff selected according to the upper arm circumference. The first reading was taken after the child had been sitting quietly for at least five minutes. The second reading was taken later the same day, towards the end of the assessment, again after the child had been sitting quietly for five minutes.

The lowest values for systolic and diastolic blood pressure were retained and compared to internationally recognised normograms previously validated in Ugandan schoolchildren [12, 13]. Children were considered to be hypertensive if the lowest systolic and/or diastolic blood pressure result was at or above the 95th centile based on their sex, age, and height. Arm span was used as a proxy for height in all children.

Renal tract ultrasound scan was performed, reporting the maximum bipolar renal length, echogenicity, and dilation of the renal collecting system. A blood sample was taken to measure creatinine, and the Schwartz formula was applied to estimate glomerular filtration rate (GFR) [14].

### 2.4. Statistical Analysis

Since only one child had elevated serum creatinine, we studied markers of renal damage that might predict future development of excretory impairment: evidence of scarring, hydronephrosis, discrepancy in renal size >1cm, and presence of hypertension. All were treated as binary variables.

The following variables were investigated for an association with evidence of renal damage, according to previously published studies: female sex, previous UTI, neurological level (motor), mobility, elevated DLPP, and CIC adherence [3, 15–21]. These were all treated as binary variables. Regarding mobility, children were categorised as either unable to walk or able to walk (with or without walking aids). A DLPP >40cm H_2_O is considered to predict an unsafe bladder, but some authors have used a threshold of >30cm H_2_O [20, 21]; therefore we investigated both for associations with renal damage.

The Chi-Square test, and Fisher's exact test where appropriate, was used to compare relationships between categorical variables. All analyses were performed using SPSS (version 23 IBM Corp.).

## 3. Results

### 3.1. Patient Characteristics

**Figure 1:**
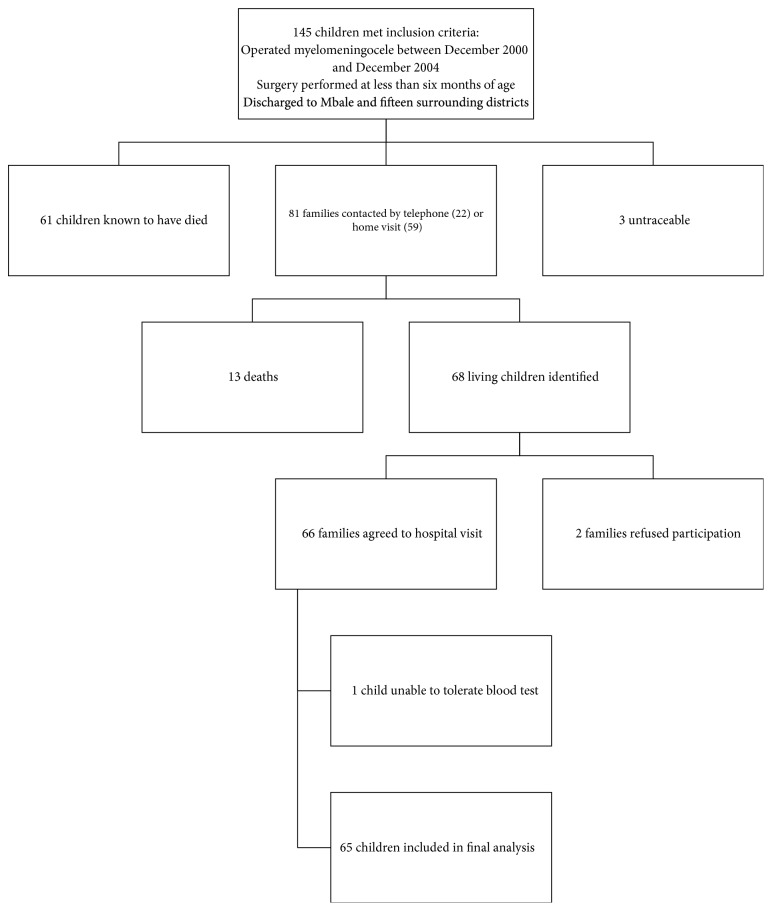
Flow diagram for patient inclusion.

Figure 1 outlines the process of patient identification and inclusion. Of 68 survivors, 66 attended CCHU for assessment. One child became distressed at the prospect of venepuncture and was therefore excluded, leaving 65 children aged 10-14 years in the final analysis.

A total of 13 children had visited the hospital within the last two years; the remainder had been lost to follow-up.

Patient characteristics are summarised in Table 1.

### 3.2. Management of Neurogenic Bladder

55 children (85%) had undergone cystometric testing on at least one occasion. Mean age at first testing was six months (range 0-45 months). The mean result for DLPP was 21cm H_2_O (range 8-56cm H_2_O). The mean DLPP among 24 females was 19.7cm H_2_O and it was 21.9cm H_2_O among 31 males (no significant difference). Four children had a DLPP greater than 40cm H_2_O. Ten children had a DLPP greater than 30cm H_2_O.

From notes review there was evidence of CIC training for parents of 60 children (92%) at a mean age of 36 months (range 0-99 months). For the five children in whom CIC was never initiated, one had a very low lesion, was continent, and underwent regular renal tract ultrasound scanning which was repeatedly normal. In the other four cases, notes review did not identify a deliberate decision, with three of the children defaulting from follow-up immediately or very soon after surgery.

At the time of this study, 17 children (26%) were undergoing CIC. Just over half (nine) were self-catheterising; CIC was performed by a caregiver in the remainder of cases. Intravesical oxybutynin was being used by six children. Of the 43 who had abandoned CIC, reasons given included running out of catheters, lack of time, and the child's distress (notably those in whom CIC had been initiated at an older age). Even among those who were performing CIC at the time of the study, notes review suggested that the majority had historically been inconsistent for similar reasons.

Only one child had undergone surgical intervention for neurogenic bladder: vesicostomy at the age of 13 months. Bladder repair and closure of suprapubic fistula were undertaken four years later.

### 3.3. Urinary Incontinence

11 children (17%) were described as “always dry” or “mostly dry,” while 54 (83%) were “always wet” or “mostly wet.” There was a strong association between urinary continence and the practice of CIC: eight of 17 children (47%) performing CIC and three of 48 (6%) not performing CIC were dry or mostly dry (*p *= 0.001). All three children who were dry and not performing CIC had a motor level at S2 or lower. Boys were more likely than girls to be incontinent of urine (92% versus 72%,* p* = 0.05), though CIC adherence between the two sexes was not significantly different.

### 3.4. Historical Urinary Tract Infections

From review of the medical records, 36 children (55%) had experienced at least one urinary tract infection (UTI); only eight children (12%) had two or more documented urinary tract infections.

### 3.5. Height and Weight

In 37 children (57%) it was not possible to measure height or recumbent length due to joint contractures, muscle weakness, or scoliosis. We therefore elected to use arm span as a proxy for height in all children. For the 28 children in whom values for both height and arm span were obtained, arm span overestimated height by a mean of 4% (range -2% to +13%). Only five children (8%) were at or above the 50th centile for height. 34 children (52%) were below the 1st centile for height. 15 children (23%) were at or above the 50th centile for BMI-for-age. 20 children (31%) were below the 1st centile for BMI-for-age.

### 3.6. Blood Pressure

28 children were hypertensive (43%). Since our results suggested that arm span overestimated height in this group, this would tend to underestimate the severity of hypertension. However, since spina bifida is associated with short stature this might overestimate the prevalence of hypertension. Recategorising blood pressure based on an assumption of height at the 50th centile for age for all children, 25 children (38%) were considered to be hypertensive, Table 2.

### 3.7. Creatinine and Estimated GFR

Creatinine was below the limit of detection for the assay (0.05mg/dL) in 27 children. Nonambulant children were overrepresented in this group, with 21/30 (70%) having a very low creatinine, compared to 6/35 (17%) of children who were ambulant with or without walking aids (*p* < 0.001).

The median creatinine for the group was 0.2mg/dL. Only one child (aged 13 and weighing 13.2kg) had an elevated creatinine at 2 mg/dL (estimated GFR of 35 ml/min/1.73m^2^). She had been identified as having a high risk bladder (DLPP 49cm H_2_O) when she initially presented for surgical repair at the age of four months. Adherence with CIC had been variable, and she had been admitted to CCHU on five occasions with urosepsis.

### 3.8. Renal Tract Ultrasound

All children underwent an ultrasound scan of the renal tract during the study visit. There was evidence of mild or moderate hydronephrosis in ten children (15%); changes were bilateral in four.

In 42 children (64%) there was increased renal cortical echogenicity, in keeping with scarring. This was graded in severity: grade 1 (mild) in 22 (52%), grade 2 in 18 (43%), and grade 3 (severe) in two (5%). In most cases the changes were bilateral; in 15 children with asymmetrical abnormalities results were classified according to the more severely abnormal kidney.

A discrepancy in maximum bipolar length between left and right kidneys of greater than 1cm was documented in 28 children (43%).

### 3.9. Risk Factors for Renal Damage

None of the 20 children with a best motor level at S1 or below had hydronephrosis on renal tract ultrasound scan, compared to 22% (10/45) of those with higher motor levels (p = 0.025). However, there was no association between motor level and scarring, discrepancy in renal size, or hypertension. Neither was there any association between mobility and evidence of renal damage (scarring, hydronephrosis, discrepancy in renal size greater than 1 cm, and presence of hypertension).

Unexpectedly, children who were documented as having at least one UTI were less likely to be hypertensive (p = 0.049). Otherwise, there was no association between any other risk factors (female sex, history of UTI, elevated DLPP, and CIC adherence) and any of the outcome measures (Table 3).

## 4. Discussion

Individuals with spina bifida are at risk of CKD as a consequence of elevated detrusor pressures, vesicoureteric reflux, and recurrent urinary tract infections, leading to renal scarring. End-stage renal disease rarely develops in childhood [11, 22] but has been reported in low-income settings and has been attributed to suboptimal management, including lack of follow-up and failure to initiate CIC with anticholinergic therapy [23, 24].

In this study we evaluated renal outcomes in a cohort of 65 Ugandan children aged 10-14 who had undergone spina bifida repair before six months of age. Urological management was suboptimal, with poor attendance at follow-up and only 17 children (26%) continuing with CIC. Over half of the original cohort were no longer alive (74 of 142 children traced).

### 4.1. Evidence of Renal Damage

The pitfalls of creatinine measurement in individuals with spina bifida, most of whom have reduced muscle mass, are well-recognised [2, 11, 15]. Furthermore, many of these Ugandan children are likely to have been undernourished. In keeping with this, we found that creatinine was below the limit of detection for the assay in 27 children (42%); the majority of these children were nonambulant, suggesting higher lesion levels and consequently reduced muscle mass. Only one child had significant renal impairment defined by eGFR. Ultrasound revealed evidence of scarring in 64% of children and a size discrepancy of more than a centimetre between kidneys in 43%. These changes are consistent with irreversible cortical loss.

Ultrasound is thought to be adequately sensitive for detecting clinically significant renal parenchymal defects [22, 25]. Discrepancy in renal length detected on ultrasound has been found to correlate with abnormal dimercaptosuccinic acid (DMSA) findings in both adult and paediatric populations [26, 27].

In this cohort, 25 children were found to be hypertensive (38%) based on two readings during a single visit. Hypertension is well-recognised among children and young adults with spina bifida [28, 29] and is associated with progression of CKD, as in adults [2]. Treatment of hypertension in a low-income setting is challenging.

### 4.2. Risk Factors for Renal Damage

Many studies have investigated predictors of poor renal outcomes among patients with spina bifida in high-income countries. In the prospectively followed Cambridge cohort, 22 of 78 deaths (28%) at a mean age of 46 years were attributed to urological causes. Deaths from urological causes occurred only in those with a sensory level of L1 or above, and there was only one urological death in those with motor level of L2 and below (neurological level assessed at birth) [3]. A cross-sectional study of 120 adults in the Netherlands found that ambulant patients (as opposed to wheelchair users) were unlikely to have unfavourable urodynamic findings [16].

The anatomical explanation for these findings is not obvious, bearing in mind that the innervation of the bladder and urethral sphincter extends as far as S2-S4 [30]. Certainly our results are not so reassuring. While none of the children with a low spinal lesion (motor level S1 and below) had hydronephrosis, there was no significant difference in the incidence of renal scarring, size discrepancy, or hypertension between this group and those with higher motor involvement. Furthermore, there was no correlation between ambulatory status and any of the markers of renal damage. Therefore, low neurological level should not lead to complacency in follow-up.

Our previously published survival study did identify an increased hazard of death among children with motor level L2 and above. Information relating to cause of death was available for 45/58 (78%) children. There was one death due to pyelonephritis (in a child aged 13 months with motor level L1) but no other urological causes were identified [7]. However it is most likely that parents would be unaware of the existence of CKD, and even end-stage renal failure, particularly since the majority of children were not attending follow-up.

Many studies have found an association between various urodynamic parameters and renal damage, though the predictive value of DLPP alone has not always been demonstrated [15, 17–20]. Elevated DLPP (using a cut-off of either >30cm H_2_O or >40cm H_2_O) measured at a mean age of six months was not found to be associated with renal scarring, kidney size discrepancy, hydronephrosis or hypertension in our cohort. More sophisticated urodynamic testing to formally describe the bladder pathology is not feasible in this setting; a decision to commence CIC based on DLPP results is inadequately sensitive and therefore unsafe.

In this cohort, children with a history of one or more urinary tract infections (UTIs) were less likely to be hypertensive. It is difficult to explain this finding. Some studies have found an association between UTIs during childhood and renal deterioration in later life [15]. Female sex has been identified as a risk factor for renal deterioration [17, 18], but we did not find this association in our relatively small cohort.

Delayed initiation of CIC, late referral, and poor adherence with treatment have all been associated with renal scarring, and the relationship with complex social circumstances has also been noted [17, 18, 20]. These issues are particularly relevant in our patient population and highlight the crucial need for education and support of families of a child with spina bifida.

### 4.3. Urinary Incontinence

Irrespective of renal tract damage and the development of CKD, urinary continence is an important goal of therapy for patients with neurogenic bladder. Incontinence has been reported to negatively affect quality of life and self-esteem [4, 5]. Two-thirds of patients can achieve social continence with CIC and anticholinergic medication [8], yet the majority of these children (83%) were described as “always wet” or “mostly wet.”

The consequences of urinary incontinence will be amplified in a low-income setting, where the cost of nappies is prohibitive, and where laundry and personal care are more challenging. Indeed, we found that urinary incontinence was associated with poorer self-reported quality of life scores in this cohort of children [4]. This issue is also relevant to school attendance in Uganda [31].

### 4.4. Management of Neurogenic Bladder

The initiation of CIC for this cohort was not universal, prompted by elevated DLPP, abnormalities detected on follow-up ultrasound scans, or incontinence. Of 60 children in whom CIC was initiated, the majority had abandoned the practice. Even among the 17 who were performing CIC at the time of assessment, most had been erratic historically.

The relative merits of an expectant versus a proactive approach to the high risk bladder continue to be debated [8, 9, 22, 32]. An expectant approach requires frequent follow-up and close monitoring [33]. It also assumes that upper tract deterioration is reversible in all patients and does not address bladder compliance changes which may occur very early.

In a low-income setting, limited investigations are available and cost considerations significant. Attendance at long-term follow-up is generally poor. Urological surgery is unaffordable for the majority of families. Later institution of CIC can be difficult for the older child, and the family, to adapt to.

CCHU is one of a very small number of centres throughout Africa managing large numbers of children with spina bifida, supported by IFSBH. Current practice involves early institution of CIC (five times daily) with intravesical oxybutynin in all children following spina bifida repair. Subsequent treatment is tailored according to ultrasound findings and the development of safe urinary continence [22, 34]. While this is regarded as optimal medical management of the neurogenic bladder, outcomes from this cohort raise serious concerns about long-term adherence. In response to the findings of this study, a Spina Bifida Specialist Nurse was appointed in 2015, and the team expanded to include two nurses in 2017. We hope to demonstrate that a long-term relationship between families and an experienced member of staff, established during the first admission, will improve attendance at follow-up and adherence with treatment.

### 4.5. Limitations

Since this is a retrospective study, our findings are limited by the strong possibility of survival bias. We did not have complete information regarding cause of death, and in most cases we would expect the existence of CKD to be unknown, even if it contributed to or caused death. We did not reproduce findings from other studies in relation to risk factors for renal deterioration. This may, in part, be due to the relatively small size of the cohort.

We were unable to measure height in 57% of our subjects, and therefore we substituted arm span for all children. Arm span is frequently used to monitor growth in children with spina bifida, although the limitations of using it in place of height are well-recognised [35–37]. The study design did not allow for repeat measurements of blood pressure at follow-up visits as advised by international guidelines [12].

## 5. Conclusions

We have demonstrated that incontinence, ultrasound evidence of renal damage, and hypertension are highly prevalent in a cohort of Ugandan children surviving at least ten years after spina bifida repair, including those children with low neurological lesions (S1 and below) and previously normal DLPP. When treatments such as urological surgery, antihypertensive medication, dialysis, and transplantation are unavailable, prevention is of even greater importance.

Our findings support the early and universal initiation of CIC with anticholinergic therapy in this setting. Enabling families to persevere with this and supporting the transition to self-catheterisation should be a priority.

## Figures and Tables

**Table 1:** Patient characteristics.

	**Male (total 36)**	**Female (total 29)**
	**n (**%**)**	**n (**%**)**
Mean age at assessment (months)	141	146
Current motor level		
L2 and above	5 (14)	2 (7)
L3-L4	15 (42)	16 (55)
L5 and below	16 (44)	11 (38)
Mobility		
Able to walk (with or without aids)	20 (56)	15 (52)
Unable to walk	16 (44)	14 (48)
Detrusor leak point pressure (available for 55 children)		
Greater than 30cm H_2_O	5 (14)	5 (17)
Greater than 40cm H_2_O	3 (8)	1 (3)
Currently performing CIC		
Yes	7 (19)	10 (35)
No	29 (81)	19 (66)
Continence		
Always dry or mostly dry	3 (8)	8 (28)
Always wet or mostly wet	33 (92)	21 (72)
Previous symptomatic culture positive urinary tract infection		
None documented	17 (47)	12 (41)
At least one	19 (53)	17 (59)
Hypertension (based on height 50th centile)		
Yes	15 (42)	10 (35)
No	21 (58)	19 (66)
Renal scarring on ultrasound scan		
Normal size, shape and echogenicity of both kidneys	15 (42)	8 (28)
Echogenic grade 1 (at least one kidney)	12 (33)	10 (35)
Echogenic grade 2 (at least one kidney)	9 (25)	9 (31)
Echogenic grade 3 (at least one kidney)	0 (0)	2 (7)
Discrepancy in kidney size on ultrasound scan		
Less than 1cm	21 (58)	16 (55)
Greater than 1cm	15 (42)	13 (45)
Hydronephrosis on ultrasound scan		
Yes	5 (14)	5 (17)
No	31 (86)	24 (83)

**Table 2:** Frequency of hypertension. Hypertension was defined as lowest systolic and/or diastolic blood pressure at or above the 95th centile based on age and height.

	Frequency using arm span as proxy for height (%)	Frequency assuming height at 50^th^ centile for age (%)
Hypertensive	28 (43)	25 (38)

Not hypertensive	37 (57)	40 (62)

**Table 3:** Risk factors for renal damage.

	**Markers of renal damage**			
**RISK FACTORS**	**Hydronephrosis**	**Renal scarring**	**Kidney size discrepancy >1cm**	**Hypertension**
**Motor level**				
L5 or above	**10/45**	30/45	20/45	18/45
S1 or below	**0/20**	12/20	8/20	7/20
	**(p = 0.025)**	(p = 0.60)	(p = 0.74)	(p = 0.70)

**Mobility**				
Unable to walk	7/30	19/30	12/30	13/30
Able to walk (with or without aids)	3/35	23/35	16/35	12/35
	(p = 0.17)	(p = 0.84)	(p = 0.64)	(p = 0.46)

**Sex**				
Female	5/29	21/29	13/29	10/29
Male	5/36	21/36	15/36	15/36
	(p = 0.74)	(p = 0.24)	(p = 0.80)	(p = 0.55)

**Urinary tract infection**				
At least one prior UTI	5/29	23/36	17/36	**10/36**
No documented UTI	5/36	19/29	11/29	**15/29**
	(p = 0.74)	(p = 0.89)	(p = 0.45)	**(p = 0.05)**

**CIC adherence**				
Never performed or abandoned CIC	6/48	30/48	22/48	18/48
Currently performing CIC	4/17	12/17	6/17	7/17
	(p = 0.43)	(p = 0.55)	(p = 0.45)	(p = 0.80)

**Detrusor leak point pressure** ^**#**^				
DLPP >30	1/10	6/10	6/10	5/10
DLPP <30	8/45	31/45	18/45	17/45
	(p = 1.0)	(p = 0.71)	(p = 0.30)	(p = 0.50)

UTI = urinary tract infection; DLPP = detrusor leak point pressure; ^#^ DLPP results available for 55 children.

## Data Availability

The data used to support the findings of this study are available from the corresponding author upon request.
